# Process-evaluation alongside a cluster-randomized trial examining the effectiveness of the ‘SELF-program’ on nurses’ activity encouragement behavior

**DOI:** 10.1016/j.ijnsa.2026.100525

**Published:** 2026-03-16

**Authors:** Michel Bleijlevens, Janneke de Man-van Ginkel, Gerard van Breukelen, Sandra Zwakhalen, Lotte Hermens, Silke Metzelthin, Getty Huisman-de Waal, Stan Vluggen

**Affiliations:** a Maastricht University, Care and Public Health Research Institute, Department of Health Services Research, Maastricht, the Netherlands; b Limburg Living Lab in Ageing and Long-Term Care, Maastricht, the Netherlands; c Radboud University Medical Center, Nijmegen, the Netherlands; d Leiden University Medical Center, Leiden, the Netherlands; e Maastricht University, Care and Public Health Research Institute and Graduate School of Psychology and Neuroscience, Department of Methodology and Statistics; f Zuyd University of Applied Sciences, Academy of Nursing, Heerlen, the Netherlands

**Keywords:** Function focused care, Process-evaluation, Nursing home staff, Nursing home residents, Medical Research Council framework, Reablement, Self-reliance

## Abstract

**Background:**

The SELF-program is an interactive, tailored and theory-based training program that aims to improve nurses’ activity encouragement behavior and indirectly to optimize nursing home residents’ self-reliance in activities of daily living. The aim of this process-evaluation study was to evaluate the implementation, mechanisms of impact and contextual factors influencing the implementation and outcomes of the SELF-program.

**Methods:**

A process-evaluation with a mixed-methods design was conducted alongside a two-arm (SELF-program vs. care as usual) cluster randomized trial of nine months, examining the effectiveness of the program in Dutch nursing home care. Nurses from various qualification levels – registered and non-registered – were eligible to participate. Guided by the Medical Research Council framework, data on implementation parameters, mechanisms of impact and contextual factors were collected using checklists, evaluation forms, (attendance) logbooks, questionnaires and focus-group interviews among nurses and trainers. Quantitative data was analysed using mixed linear regression analyses, qualitative data using a deductive coding approach.

**Results:**

Twenty-eight nursing home wards – fourteen in each condition – from three care organizations across the Netherlands were included in the trial. Nurses were highly satisfied with the program and particularly valued its interactive content and team approach. The program was implemented with high fidelity, with 90% of all sessions completed and attendance rates averaging 60%. Attendance varied between organizations and declined over time. Improvements were observed in nurses’ attitudes, perceived social influence, self-efficacy beliefs and intentions towards performing activity encouragement behavior, of which most were statistically significant, corresponding with small to medium effect sizes. These results were endorsed by data derived from focus-group interviews with trainers and nurses. Still, time constraints, staffing shortages and lack of motivation were put forwards as factors leading some nurses to still take over tasks. The presence of organizational policy regarding activity encouragement behavior, family member support, motivation of nurses, involving other disciplines and the interactive approach facilitated program implementation and outcomes. Contextual barriers to program implementation and outcomes included staffing shortages, low program attendance, time constraints, lack of manager support, and delivery of the program during the COVID-19 pandemic.

**Conclusions:**

Overall, the SELF-program was well received and implemented, with favourable improvements in mechanisms known to impact nurses’ activity encouragement behavior. The program and its implementation could benefit from minor adjustments, as well as an improved attendance. Considering the results of this thorough process-evaluation as well as the positive results of the program on nurses’ activity encouragement behavior, a widespread implementation is recommended.

**What is already known**

• Nurses play a crucial role in encouraging activity and independent functioning of older people, however, they often take over tasks unnecessarily and habitually.
• The SELF-program is an interactive, holistic, tailored and theory-based program that aims to improve activity encouragement behavior in nurses and recently its effectiveness was demonstrated through a cluster-randomized trial.
• Process-evaluations are scarce but crucial in understanding underlying processes and merely investigating an intervention’s effectiveness overlooks information on how and why a program works (or not) and could be optimized.

Alt-text: Unlabelled box dummy alt text

**What this paper adds**

• The SELF-program was implemented with high satisfaction and fidelity, achieving 90 % session completion and an average attendance rate of 60 %, though attendance varied across organizations and declined over time.
• The SELF-program resulted in improvements in nurses’ attitudes, perceived social influence, self-efficacy beliefs and intentions towards performing activity encouragement behavior, of which most were statistically significant, corresponding with small to medium effect sizes.
• Contextual facilitators and barriers of the implementation and outcomes of the SELF-program were exposed. Facilitators included the presence of organizational policy regarding activity encouragement behavior, family member support, motivation of nurses, involving other disciplines and the interactive program approach. Barriers included time constraints, low program attendance, staffing shortages, lack of manager support and COVID-19 respectively.

Alt-text: Unlabelled box dummy alt text

## 1 Introduction

Worldwide, many countries are dealing with ageing populations (United Nations, 2019). Likewise, in the Netherlands, it is expected that over a quarter of the total population will be 65 years or older by 2060 (CBS, 2020). The demographic transition is accompanied by societal and financial challenges and poses a direct threat to people’s functional capabilities. At a certain point in life, people may develop dependencies and consequently they may require care support to complete tasks fundamental to daily living. The government’s ageing-in-place policy helps older adults to live in their familiar surroundings for as long as possible, ensuring a better quality of life (Rijksoverheid, 2018; Janse et al., 2018). However, some people may require long-term care in nursing homes where round-the-clock care support is offered for people with increased somatic or psychogeriatric frailty (Verbeek-Oudijk and Koper, 2021; Mendes et al., 2016). Despite their compromised capabilities, it is crucial for nursing home residents to stay active and self-reliant, in order to live an autonomous and dignified life (WHO, 2020). However, internationally it is widely acknowledged that residents spend most of their day in an inactive – sitting or lying – position, independent of their care background (den Ouden et al., 2015). Innovative care approaches are required to turn this trend around.

In the Netherlands, care support is generally offered by nursing staff throughout the entire care landscape. According to Henderson, the universal element in the concept of nursing is to work towards a ‘healthy’ independence of the individual (Henderson, 1978). As nurses spend the most time with residents compared to any other discipline, they have a key role to play in encouraging and enabling residents to improve their functional capabilities and independence (Westbrook et al., 2011). However, the focus of care is mostly task-oriented and meeting the medical needs of residents rather than optimizing their functional health, thereby facilitating further functional decline and care dependency (Resnick et al., 2009). Observational research conducted in Dutch nursing homes demonstrates that nurses provided support or took over activities of daily living of residents in over 95 % of the instances, in contrast to observing and interfering only when necessary (den Ouden et al., 2017). This is even more worrisome as ageing people generally strive for improvements or independence in these kind of self-care activities, such as mobility and personal care (Mouchaers et al., 2024). Nevertheless, nurses do feel they have a responsibility in enhancing functional capabilities of residents, although various barriers e.g., on resident, nurse, environmental and organisational level may hamper them in enacting activity encouragement behavior (Swoboda et al., 2020; Resnick et al., 2008; Resnick et al., 2006; Benjamin et al., 2014). Moreover, the dire shortages within nursing staff in Western society, emphasizes the urgency to foster functional capabilities and self-reliance in older people. Clearly, nurses require support to do so.

In an effort to improve nurses’ activity encouragement behavior, restorative care approaches like Function-Focused Care and Reablement are gaining ground worldwide (Resnick et al., 2013). With the aim of supporting nurses to deliver care in which activity and independent functioning of individuals is optimized, these approaches have been the basis for many programs in different care settings worldwide. However, such programs demonstrated equivocal results regarding improvements in nurses’ activity encouragement behavior and resident outcomes (Buma et al., 2022; Lee et al., 2019). A thematic synthesis study of various Dutch and internationally developed programs provided valuable lessons and implications to improve future programs (Vluggen et al., 2021). These acquired lessons formed the basis for the development of a renewed and refined training program; the ‘SELF-program’: a holistic, theory-grounded and tailored training program, aiming to improve nurses’ activity encouragement behavior and indirectly to optimize self-reliance in activities of daily living in nursing home residents.

The effectiveness of the SELF-program has lately been examined in a large-scale cluster-randomized trial in Dutch nursing home care (Vluggen et al., 2022). The program was effective in improving nurses’ activity encouragement behavior directly after (*d* = 0.53; *p* = .003; 95 % CI 1.88 – 8.02) as well as six months after program completion (*d* = 0.38; *p* = .02; 95 % CI 0.67 – 7.27). A trend was observed towards a less pronounced decrease in self-reliance in activities of daily living in those residents allocated to wards that exposed nurses to the SELF-program compared to those receiving care as usual (Vluggen et al., 2024). However, as an evaluation goes beyond the question of whether an intervention is effective, an extensive process-evaluation was conducted alongside the trial. Process-evaluations are crucial in understanding underlying processes and merely investigating an intervention’s effectiveness overlooks information on how an intervention is implemented, and how and why an intervention works (or not) and could be optimized (Grant et al., 2013; Lockwood et al., 2022). The Medical Research Council framework provides guidance for the development, implementation and evaluation of complex interventions in a cyclical process and has been used frequently to design process-evaluation studies (Skivington et al., 2024; Kletter et al., 2025). It specifically offers structured guidance in designing process-evaluations by exploring implementation parameters, mechanisms of impact and contextual factors facilitating or hindering the implementation and outcomes of interventions through both qualitative and quantitative research methods (Skivington et al., 2021). The framework was deemed suitable for this study in preference to more implementation- or scale-up-oriented frameworks, as it is specifically designed for evaluating complex interventions across multiple clusters and uniquely integrates process-evaluation parameters directly linked to the effect evaluation.

Therefore, the aim of this process-evaluation study was to examine the implementation, mechanisms of impact and contextual factors influencing the implementation and outcomes of the SELF-program. Results will be used to refine future implementation and content of the SELF-program.

## 2 Method

### 2.1 Study design

This process-evaluation was conducted alongside a two-arm (SELF-program vs. care as usual) cluster-randomized trial in the Dutch nursing home care. According to the guidance provided by the Medical Research Council framework, both qualitative and quantitative measures should be used in conducting a thorough process-evaluation, hence a mixed-methods study design was applied (Skivington et al., 2024). The qualitative research part adhered to the elements of the Consolidated Criteria for Reporting Qualitative research, in order to improve reliability of the study (Tong et al., 2007).

### 2.2 SELF-program

**Fig. 1:**
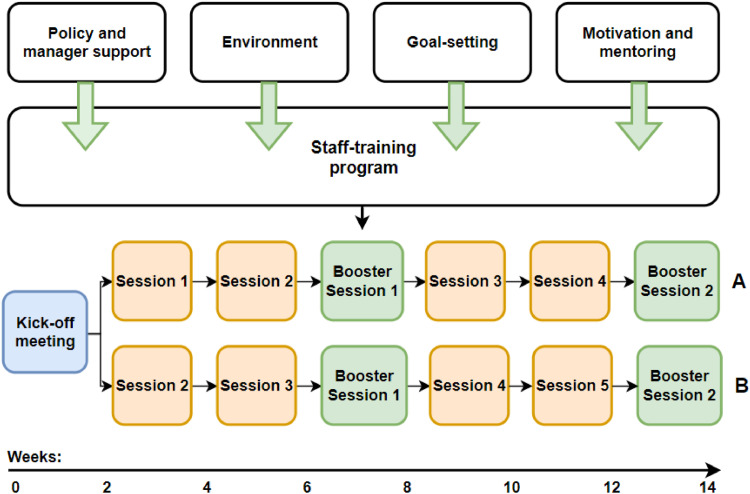
schematic overview of the SELF-program.

‘SELF’ is a Dutch acronym for self-reliance, autonomy, life quality, and functionality. Its core is a holistic, tailored staff-training program that addresses organizational policy, manager support, the physical and social environment, goal-setting, and continuously motivating both nurses and residents regarding activity encouragement behavior. The training program includes seven interactive face-to-face sessions over 14 weeks (see Fig. 1), each lasting two hours – except booster sessions, which last one hour – and uses various interactive methods.

The primary target audience of the SELF-program are nursing staff involved in providing care for activities of daily living. In the Netherlands, several nursing qualification levels can be distinguished depending on their duration of training and subsequent responsibilities, including nurse aides, nurse assistants, certified nurse assistants, vocationally-trainer registered nurses and baccalaureate-educated registered nurses. As all these nursing levels are involved in providing care for activities of daily living, all were eligible to take part in the SELF-program, hereafter referred to as ‘nurses’.

The program follows a sequential dual behavior change process for nurses, first aiming to increase their awareness, motivation, and willingness to promote self-reliance and activity in residents, then equipping them with techniques to foster this in residents. This approach is based on the Integrated Change Model, a framework often used for understanding and influencing behavior (30). The model identifies four key mechanisms impacting behavior—attitudes, perceived social influences, self-efficacy beliefs, and intention to perform activity encouragement behavior —which mediate behavior change and serve as the main targets of the SELF-program (van Sambeek et al., 2023).

The SELF-program starts with a kick-off session to raise awareness of activity encouragement behavior among nurses and assess the care team’s willingness to improve their activity encouragement behavior. After the kick-off session, a tailor-made trajectory is available for teams who are either sufficiently or insufficiently aware and willing to improve their activity encouragement behavior. A key lesson derived from previous program’s is that a one-size-fits-all approach does not achieve intended effects and that programs need to be tailored to nursing teams’ preferences to generate greater cognitive processing of its content (Vluggen et al., 2021). Therefore, if awareness and willingness are perceived to be insufficiently present, the team starts with session 1 (trajectory A); if sufficient, the team starts with session 2 (trajectory B), see Fig. 1. Based on opinion of the team manager, the concerned trainer, and nurses themselves, a decision is made on which trajectory would be most appropriate. Both trajectories are equal in duration and intensity. However, the trajectories differ in such a way that teams who enter trajectory A initially receive session 1 (and those in B do not), while participants who initially enter trajectory B receive session 5 at the end (and those in A do not); all other sessions are similar. Trajectory A primarily pays more attention to the required behavior change in nurses, while trajectory B transitions faster to the part where nurses are taught techniques to induce a behavior change in residents trajectory B more quickly focuses on techniques to induce a behavior change in residents. In session 4, nurses practice these skills through actor-guided interactions. Both trajectories are equal in duration and intensity. Booster sessions review previous content to prepare teams for upcoming sessions. A training expert from the concerned care organization leads the sessions with support from a detailed manual and a co-trainer from each ward. A full description of the program and its theoretical basis is provided elsewhere (Vluggen et al., 2022).

### 2.3 Sample and procedure

Care organizations were recruited through a national campaign promoting the SELF-program via national conferences, (social) media, and partners affiliated to the Limburg Living Lab in Ageing and Long-Term Care (Verbeek et al., 2020). A research protocol was compiled which served as the foundation for the sampling procedure (Vluggen et al., 2022). For being eligible, organizations had to provide nursing home care, independent of this being for residents with somatic, psychogeriatric or mixed care backgrounds. Care organizations usually consist of multiple sites, which in turn consist of several wards, of which the latter was chosen as unit of intervention. Which wards would participate in the trial was decided in agreement with the board, and site and ward managers. Within an organization, dyads were created which matched two wards based on their size and type (e.g., somatic, psychogeriatric or mixed). Of a dyad, one ward was randomly allocated to the intervention group and the other to the control group. To minimize the probability of contamination bias, dyads were created of wards that did not collaborate in daily practice. If it was likely that there would be collaboration between wards, something likely to occur within nursing homes in the Netherlands, randomization took place at site level. This would mean that the entire site was included in a dyad with a pair-matched comparator site of which one site including its wards was randomly allocated to the SELF-program and the other to care as usual. Randomization of either wards or sites was conducted by means of computer-randomization using random.org. The unit of intervention was the nursing home ward, i.e. the SELF-program, was offered to wards separately, independent of whether randomization took place on ward or site level. For full details on the sampling procedure, see Vluggen et al., (Vluggen et al., 2024).

### 2.4 Data collection

Process-evaluation data was collected regarding implementation, mechanisms of impact and contextual factors influencing the implementation and outcomes of the SELF-program. Qualitative and quantitative data was collected among program participants (nurses) and program (co)-trainers, before, during and after program implementation, see Table 1.

**Table 1:** overview of data collection for process-evaluation.

Process-evaluation component	Source	Data-collection method	Time points
**Implementation**			
*Dose satisfaction*	Nurses	Self-administered questionnaire^a^ (4 items) and focus-group interviews	During and after implementation
Satisfaction with program (delivery)			
*Dose delivered*	Trainers	Logbook including checklists and focus-group interviews	During and after implementation
Quantity of delivery			
*Fidelity*	Trainers	Logbook including checklists and focus-group interviews	During and after implementation
Delivery according to plan			
*Adaptations*	Trainers	Logbook and focus-group interviews	During and after implementation
Alterations made during implementation			
*Reach*	Nurses	Attendance lists and focus-group interviews	During and after implementation
Extent to which target group was exposed to program			
**Mechanisms of impact**			
Mechanisms assumed to impact outcome behavior	Nurses	Questionnaire^b^ (31 items)	Baseline (T0), 3 months after baseline (T1) and 9 months after baseline (T2)
		Focus-groups interviews	After implementation
**Contextual factors**			
Barriers and facilitating factors that may influence implementation and outcomes	Trainers and Nurses	Logbook and focus-group interviews	During and after implementation

a Numeric rating scale and open space items

b Ordinal 5-point Likert-Scale

Qualitative data was collected through focus-group interviews conducted via Zoom between July 2021 and March 2022. All participating nurses and trainers were invited by email after program completion, and interviews were scheduled with those willing to join. Researchers SV, GHW and JMG conducted the interviews, obtaining verbal consent for participation and audio recording in advance. Demographic characteristics (age, gender, professional level, and working experience) were collected, and interviews were guided by a topic list consisting of approximately ten main questions and some sub questions based on the parameters from the Medical Research Council’s process-evaluation framework (Skivington et al., 2024).

#### 2.4.1 Implementation

Implementation is related to two key questions: 1) how was the program implemented, and 2) what was the quality and quantity of what was implemented. To answer these questions, implementation can be divided into dose satisfaction, dose delivered, fidelity, adaptions, and reach (Moore et al., 2015).

*Dose satisfaction* answers the question whether nurses were satisfied with the program. After each training session, nurses’ satisfaction was assessed using a self-administered questionnaire consisting of four items: 1) a numeric rating scale to rate the concerned session ranging from 1 *‘very bad’* to 10 *‘very good’,* 2) an open space for positive aspects of the concerned session, 3) an open space for negative aspects of the concerned session, and 4) an open space for additional comments or points of improvement. The opinion of nurses regarding the SELF-program was further assessed by means of focus-group interviews conducted after program completion. *Dose delivered* provides an answer to the question of how much of the program was delivered by the (co)-trainers and thus received by the nurses. Actual delivery of the sessions and its components was measured using a logbook including checklists, to be filled in by the trainer after each session, and focus-group interviews. *Fidelity* answers the question whether delivery of the program went according to plan. The logbooks and checklists kept by the trainers were consulted to assess whether program implementation had been carried out according to protocol. Fidelity was further assessed in focus-group interviews with trainers. *Adaptations* relates to whether and if so, alterations were made to the program during implementation. Logbooks kept by trainers were consulted and focus-group interviews were conducted to assess what changes the trainers had made during the implementation process and why. *Reach* answers the question to what extent the intended target group was exposed to the intervention. Attendance lists were kept by the trainers and focus-group interviews were conducted to assess the number of people who attended the sessions including reasons for absence.

#### 2.4.2 Mechanisms of impact

To assess whether the mechanisms believed to explain behavior change in nurses’ activity encouragement behavior were impacted by the SELF-program, questionnaires and focus-group interviews were used among nurses. The questionnaire was administered before implementation of the SELF-program (baseline; T0), directly after program implementation (three months after baseline; T1) and nine months after baseline (T2). The informed consent procedure was equal to the one described in the effect-evaluation article, as the mechanisms of impact questionnaire was part of the whole data-collection procedure (Vluggen et al., 2024). The questionnaire, based on the work of Resnick & Simpson and Sambeek et al., consisted of 31 items, assessing nurses’ attitudes, perceived social influence, self-efficacy beliefs and intention to perform activity encouragement behavior (Resnick and Simpson, 2003; van Sambeek et al., 2023). *Attitude* was measured with 11 items on an ordinal 5-point Likert scale from 1 (totally disagree) to 5 (totally agree) and assessed whether the nurses agreed with statements such as e.g., *‘Applying activity-encouragement behavior gives me the feeling that I am providing important care’. Social influence* was measured with eight items on an ordinal 5-point Likert scale from 1 (totally disagree) to 5 (totally agree). Nurses were asked to rate their level of agreement with statements such as e.g., *‘My colleagues provide a good example of how to apply activity encouragement behavior’. Self-efficacy* was measured with 11 items on an ordinal 5-point Likert scale from 1 (no confidence) to 5 (total confidence). Nurses were asked to what extent they agreed with statements such as: how much confidence do you have that you are able to… e.g., *‘actively involve residents in the washing process?’ Intention* was measured with one item, on an ordinal 5-point Likert scale from 1 (totally disagree) to 5 (totally agree) to assess the extent to which nurses agreed with the statement, ‘*I am willing to implement activity encouragement behavior (more) with my residents.’* Additional to the questionnaire, focus-group interviews were conducted to gain a deeper understanding of which and how these mechanisms were impacted by the SELF-program.

#### 2.4.3 Contextual factors

Contextual factors may influence the implementation and outcomes of the SELF-program in a positive or negative manner. To assess if, which and how such factors played a role, logbooks were kept, and focus-group interviews were conducted with trainers and nurses.

### 2.5 Data analysis

#### 2.5.1 Quantitative data analysis

Summary statistics (mean and standard deviation) were used to describe data from continuous variables used to assess program satisfaction. Ratings of the sessions are displayed per session, for the overall sample, per organization, and per trajectory (A or B), accompanied by the concerned number of respondents (N). Logbook and checklist data on dose delivered was used to determine the percentage of sessions that were delivered. Logbook data on reach was used to calculate the attendance rates per session, for the whole sample, per organization, and per trajectory. To describe sample characteristics of either participants in the trial or those taking part in the focus-group interviews, summary statistics (mean and standard deviation) were used for continuous variables whereas categorical variables were described using the absolute count and percentages.

Equal to the analysis of the effectiveness of the SELF-program on nurses’ activity encouragement behavior, the effectiveness of the SELF-program on mechanisms of impact was determined using mixed linear regression analyses in SPSS. A scale-score was created for each concept separately, consisting of a mean score of the separate items. First, a three-way interaction between treatment, time and organization was examined. If statistically significant, then the analysis was repeated per organization, with treatment, time and treatment by time as predictors. If no three-way interaction was found, then it was removed from the model to estimate and test the treatment by time interaction of interest on the total sample. The treatment by time interaction was plotted for each concept separately (attitude, social-influence, self-efficacy and intention), both based on the observed outcome and the outcome predicted by the regression model (unlike the former, the latter is model-dependent but adjusts for bias due to attrition). P-values, confidence intervals and effect sizes (Cohen’s *d*) as defined for mixed-regression are reported per time point. For full technical details of the analysis, see Vluggen et al. (2024).

#### 2.5.2 Qualitative data analysis

The audiotapes of the focus-group interviews were transcribed verbatim, anonymized and entered into ATLAS.ti version 9.0 (ATLAS.ti, 2022). A deductive coding approach was applied making use of the components and subcomponents derived from the Medical Research Council framework (Fife and Gossner, 2024). Researcher LH independently coded the transcripts in close consultation with researchers SV and SM. First, transcripts were read carefully to familiarize with the data while identifying and highlighting relevant text segments. After achieving agreement among the involved researchers regarding relevant text segments, these were coded under relevant components and subcomponents of the Medical Research Council framework. Subsequently, this process was repeated for each transcript, where after objective text sections were composed and linked to the components and subcomponents of the relevant framework to be presented as study results. The qualitative results are supported with literal quotes from either nurses or trainers, specifying different nurses and trainers through numerical values.

### 2.6 Ethical approval

The study is registered in the Dutch Trial Register (NL9189) as of December 2020 and was approved by the Zuyderland Medical Ethics Review Committee (METCZ20210007). Recruitment commenced in March 2021 and ended in September 2021; follow-up lasted until July 2022.

## 3 Results

### 3.1 Sample characteristics

Twenty-eight nursing home wards from eight sites and three care organizations across the Netherlands were included in the trial. The 28 wards were randomly assigned to the intervention (SELF-program, *n* = 14) or control (care as usual, *n* = 14) group, according to the pre-arranged randomization procedure (Vluggen et al., 2024). Eight intervention wards continued with trajectory ‘A’ while six proceeded with trajectory ‘B’. Table 2 presents baseline nurse characteristics by condition and overall. After program completion, six focus-group interviews were conducted, ranging in duration from 42 to 84 min, of which three with program trainers (one per organization) and three with nurses, generally a mixture of organizations. In organization 3, no focus-group interview with nurses took place, because no nurses were willing or available to take part. Regarding the three interviews with trainers, in total eight females and one male took part, with an average age of 46.4 years (SD = 14.6). Trainers worked at their organization as a registered nurse, learning expert (*N* = 3), practical trainer (*N* = 2), social worker, science practitioner and (quality) policy officer. Regarding the three interviews with nurses, in total nine nurses – only females – took part, with an average age of 46.9 years (SD = 14.5) and average working experience of 17.6 years (SD = 13.7). The sample consisted of two registered nurses, four certified nurse assistants, one nurse aide and two nurse activity supervisors.

**Table 2:** Baseline sample characteristics of nurses in the trial.

Nurses			
	Intervention (N=152)	Control (N=135)	Total (N=287)
Organization 1, n (%)	38 (25.0)	35 (25.9)	73 (25.4)
Organization 2, n (%)	65 (42.8)	70 (51.9)	135 (47.0)
Organization 3, n (%)	49 (32.2)	30 (22.2)	79 (27.5)
Age in years, mean (SD)^a^	40.4 (12.9)	41.3 (14.4)	40.9 (13.6)
Gender, female, n (%)	141 (93%)	130 (96%)	271 (94%)
Care experience in years, mean (SD)	14.0 (11.0)	13.7 (11.4)	13.9 (11.1)
Care experience in nursing home, years (SD)	9.9 (9.8)	8.4 (9.4)	9.2 (9.6)
Weekly contract hours, hours (SD)	26.9 (5.4)	26.2 (6.4)	26.6 (5.9)
Education level, n (%)			
Low	30 (19.7)	34 (25.2)	64 (22.3)
Middle	106 (69.7)	90 (66.7)	196 (68.3)
High	16 (10.5)	11 (8.1)	27 (9.4)
Professional nursing level, n (%)			
Nurse Aide	10 (6.6)	8 (5.9)	18 (6.3)
Nurse Assistant	35 (23.0)	32 (23.7)	67 (23.3)
Certified Nurse Assistant	74 (48.7)	66 (48.9)	140 (48.8)
Vocationally-Trained Registered Nurse	25 (16.4)	24 (17.8)	49 (17.1)
Baccalaureate-Educated Registered Nurse	8 (5.3)	5 (3.7)	13 (4.5)

a SD = Standard deviation

### 3.2 Implementation

#### 3.2.1 Dose satisfaction

In general, nurses were satisfied with the program, reflected by the mean scores given to each separate session, ranging from 7.4 to 8.5 on a scale from 1 to 10. The actor-guided session was rated with the highest score of 8.5. Table 3 shows the average satisfaction ratings, standard deviations, and absolute number of nurses per session, for the total sample, per organization and per trajectory. Ratings were comparable between organizations and trajectories. The qualitative data obtained from the focus-group interviews and positive aspects derived from the evaluation forms endorses these findings. Positive aspects that were mentioned most frequently included that the training created awareness, was interactive, and provided the possibility to discuss the subject openly among colleagues. In the interviews, nurses mentioned they appreciated the actor-guided session, practical application, applying the content to their own residents and the team approach, which allowed them to learn from each other’s experiences.

**Table 3:** Ratings of the sessions displayed for total sample, per organization and trajectory (A/B).

	Kick-off	Session 1	Session 2	Session 3	Booster 1	Session 4	Session 5	Booster 2
Total sample								
Mean (SD)	8.0 (.9)	7.4 (1.2)	8.1 (1.0)	7.9 (.8)	7.4 (1.2)	8.5 (.9)	8.3 (.7)	7.9 (.8)
Range	(6-10)	(4-10)	(5-10)	(5-10)	(5-10)	(6-10)	(7-10)	(7-10)
N	126	66	89	73	84	79	30	50
Organization 1								
Mean (SD)	7.9 (.8)	7.6 (.5)	8.0 (1.1)	7.4 (.9)	7.8 (.8)	8.2 (1.1)	8.7 (.8)	7.5 (.5)
N	38	7	29	24	24	26	10	15
Organization 2								
Mean (SD)	8.1 (1.0)	8.1 (.7)	8.2 (.9)	8.1 (.8)	7.9 (.9)	8.7 (.9)	8.4 (.5)	8.3 (.8)
N	59	37	46	40	40	41	14	28
Organization 3								
Mean (SD)	7.8 (.7)	6.2 (1.0)	8.0 (1.0)	8.2 (.7)	6.0 (.9)	8.1 (.7)	7.5 (.5)	7.2 (.5)
N	29	22	14	9	20	12	6	7
Trajectory A								
Mean (SD)	8.0 (.8)	7.4 (1.2)	8.0 (.9)	7.8 (.9)	7.5 (1.2)	8.6 (.9)	a	8.1 (1.1)
N	69	66	44	37	43	37		18
Trajectory B								
Mean (SD)	7.9 (1.0)	a	8.2 (1.0)	7.9 (.8)	7.3 (1.1)	8.4 (1.0)	8.3 (.7)	7.8 (.6)
N	57		45	36	41	42	30	32

a Session not part of the trajectory

*‘’Those cases we discussed, we got some tips like ‘you could approach it like this or like that’, we were able to discuss it among ourselves, so I have given my opinion, others were able to give their opinion. … [she] told me how she handles it, so when I run into the fact that I can't get anything done with that resident and she [actually] does, then I knew why it might not work for me.’’ [nurse 1]*

In general, few negative aspects were mentioned about the training sessions. The main points were too much repetition, a desire for more practical examples, and low attendance by colleague nurses. Some nurses felt the total amount of sessions was too much. The focus-group interviews with the trainers confirmed these findings. The practical assignments were clear, but some nurses viewed it as a burden because of a shortage of time or considered ‘homework’ as invaluable. In case the assignment was not prepared sufficiently, trainers dealt with this by walking through the practical assignment in the subsequent session. Furthermore, in one organization a few nurses experienced having to come back for the relative short booster sessions as a burden.

#### 3.2.2 Dose delivered

Logbook data revealed that 88 of the possible 98 sessions (90 %) were delivered. Both checklist and interview data showed that within those 88 sessions, almost all separate components were completed. Reasons for those 10 sessions not taking place included national COVID-19 measures, planning issues and illness. According to the trainers of organization 3, it was remarkable that due to multiple circumstances such as low motivation, national COVID-19 measures and being a self-managing ward, two wards assigned to trajectory A, did not complete the program. This resulted in seven missed sessions within these two wards, while in two other wards from the same organization, assigned to trajectory B, only one session was not provided.

#### 3.2.3 Fidelity and adaptations

Logbook data showed no major deviations in the delivered sessions and their components. Most of the program components were fully executed by the trainers without notable adaptations. If deviations were reported, they were mostly related to time issues. For example, in a few teams, there were resistance issues and a lack of motivation, which caused a lack of time for some components. Further, occasionally minor content adjustments were made by the trainer, for example taking more time for a certain component that appealed to the group, and less time for components that did not. Last, due to technical issues, a few videos were not shown in some sessions.

*‘’When you see that the whole group has a very nice discussions together, then I let that happen because we learn from that, and there was also the space for that in the training, you would win time in another part.’’ [Trainer 1]*

Trainers felt well-prepared to provide the program, finding the manual clear and easy to use. Those delivering both trajectories (A and B) simultaneously for various teams, initially struggled with the differing preparations, as the paths diverged after the kick-off. However, this later eased preparation since they had already delivered the sessions once. Trainers also valued the periodic intervision meetings with colleague trainers and researchers to address challenges.

*‘’The program preparation we received was complete, I thought that was very nice… everything was delivered ready-made, it was very easy to pick up.’’ [Trainer 4]*

#### 3.2.4 Reach

Average session attendance ranged from 48 to 75 %, with an overall average of 60 %. Attendance varied slightly by organization: 66 % in organization 1 (56–87 %), 62 % in organization 2 (40–78 %), and 50 % in organization 3 (27–68 %). It also differed slightly between trajectories: 51 % in A (38–73 %) and 60 % in B (48–78 %). Trainers and nurses cited vacation, illness, days off, and night shifts as main reasons for absence. Attendance was mandatory in most teams, except in organization 3. Attendance rates declined over time, with the highest at the kick-off session and lowest at the second booster session. Trainers and nurses attributed this to tight scheduling, low motivation, high workload, COVID-19 measures, and sessions scheduled during holidays. To improve attendance, they suggested online booster sessions, more interactive sessions, smaller groups, fewer sessions, longer intervals between sessions, and involving other disciplines and welfare staff for broader organizational impact.

### 3.3 Mechanisms of impact

#### 3.3.1 Quantitative results

Changes in mechanisms believed to impact nurses’ activity encouragement behavior, that is attitude, social influence, self-efficacy and intention, were analysed using mixed linear regression.

*Attitude*

**Fig. 2:**
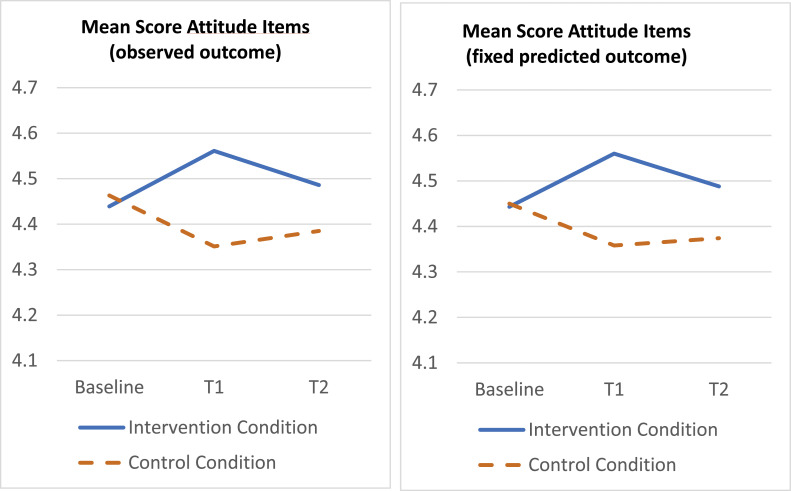
Time course of mean attitude score per treatment condition based on observed outcome (left) and fixed predicted outcome (right). Y-axis represents mean scores on 5-point Likert scale (1 = totally disagree to 5 = totally agree).

On average at baseline, nurses in the trial (totally) agreed to the items assessing their attitude towards activity encouragement behavior. Mixed linear regression of the attitude scale on treatment, time and organization plus their two-way and three-way interactions, showed no three-way interaction (*p* = .063), and after deleting the three-way term showed a borderline statistically significant treatment x time interaction (*p* = .053). Specifically, the mean outcome difference on the attitude scale between treated and control, improved with approximately 0.208 points (*p* = .016; 95 % CI 0.039 – 0.377; *d* = 0.31) at T1 and with approximately 0.121 points (*p* = .139; 95 % CI −0.039 – 0.281; *d* = 0.20) at T2, compared with the baseline difference, see Fig. 2.

*Social influence*

**Fig. 3:**
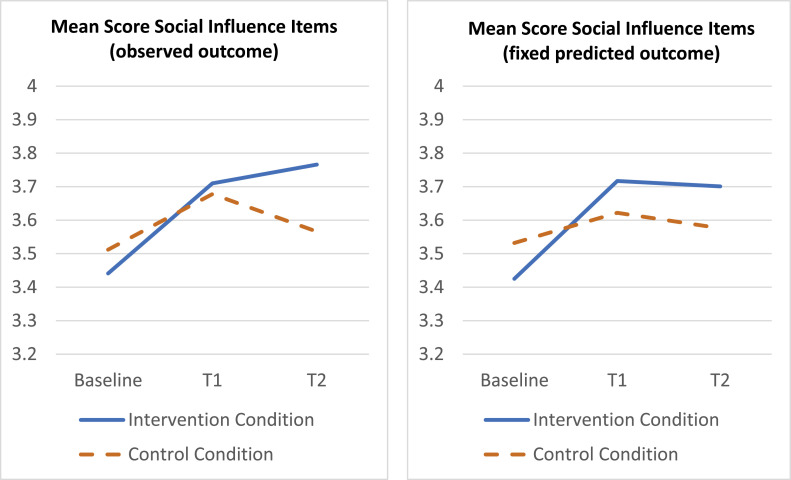
Time course of average social influence score per treatment condition based on observed outcome (left) and fixed predicted outcome (right). Y-axis represents mean scores on 5-point Likert scale (1 = totally disagree to 5 = totally agree).

On average at baseline, nurses in the trial were neutral/agreed to the items assessing their perceived social influence towards activity encouragement behavior. Mixed regression of the social influence scale on treatment, time and organization plus their two-way and three-way interactions, showed a significant three-way interaction (*p* = .025). In view of multiple outcome testing (which calls for a smaller α for significance testing than 0.05, e.g., 0.01) the results are here given based on the model without three-way interaction for simplicity. An analysis per organization is reported in the online supplement, showing that a treatment x time effect was found in organization 2 only. Based on the total sample and the model without three-way term, a statistically significant treatment x time interaction was found (*p* = .033). Specifically, the mean outcome difference on the social influence scale between treated and control, improved with approximately 0.20 points (*p* = .034; 95 CI 0.016 – 0.388; *d* = 0.28) at T1 and with approximately 0.23 points (*p* = .021; 95 % CI 0.036 – 0.428; *d* = 0.32) at T2, compared with the baseline difference, see Fig. 3.

*Self-efficacy*

**Fig. 4:**
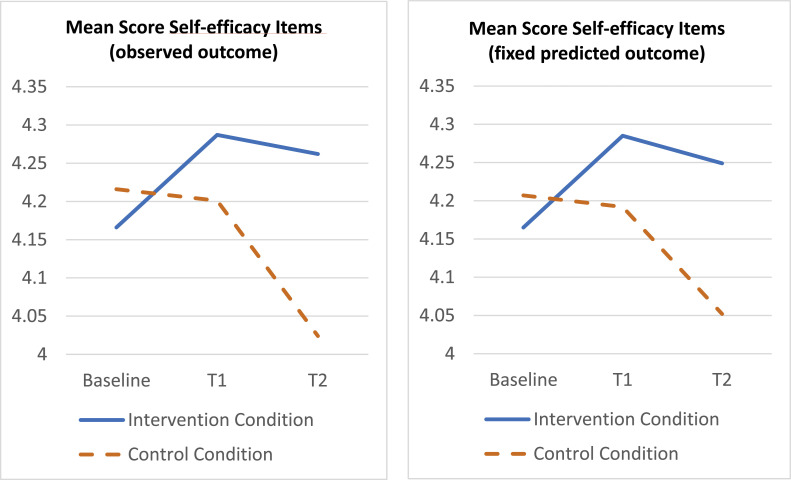
Time course of average self-efficacy score per treatment condition based on observed outcome (left) and fixed predicted outcome (right). Y-axis represents mean scores on 5-point Likert scale (1 = no confidence to 5 = total confidence).

On average at baseline, nurses in the trial felt confident to actively involve residents in various activities of daily living addressed in the items assessing their self-efficacy. Mixed regression of the self-efficacy scale on treatment, time and organization plus their two-way and three-way interactions, showed no three-way interaction (*p* = .646), and after deleting the three-way term showed a statistically significant treatment x time interaction (*p* = .026). Specifically, the mean outcome difference on the self-efficacy scale between treated and control, improved with approximately 0.135 points (*p* = .066; 95 % CI −0.009 – 2.78; *d* = 0.24) at T1 and with approximately 0.239 points (*p* = .008; 95 % CI 0.064 – 0.414; *d* = 0.37) at T2, compared with the baseline difference, see Fig. 4.

*Intention*

**Fig. 5:**
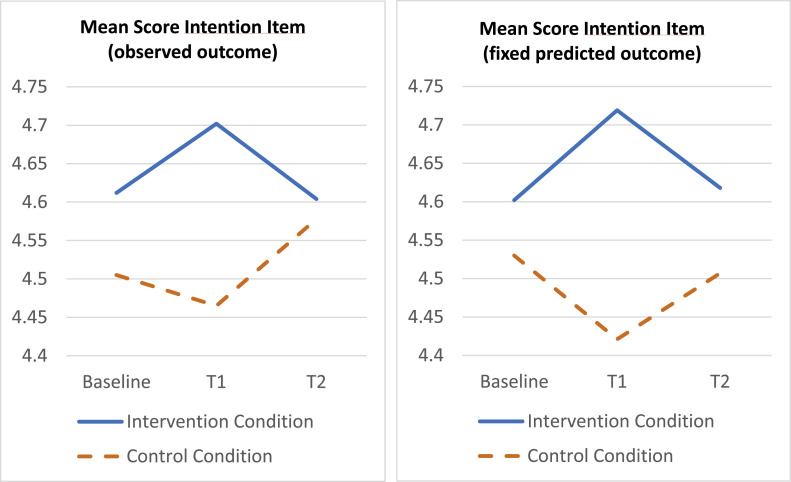
Time course of average intention score per treatment condition based on observed outcome (left) and fixed predicted outcome (right). Y-axis represents mean scores on 5-point Likert scale (1 = totally disagree to 5 = totally agree).

On average at baseline, nurses in the trial (totally) agreed to be willing to improve their activity encouragement behavior. Mixed regression of the intention scale on treatment, time and organization plus their two-way and three-way interactions, showed a statistically significant three-way interaction (*p* = .025). In view of multiple outcome testing (which calls for a smaller α for significance testing than 0.05, e.g., 0.01) the results are here given based on the model without three-way interaction for simplicity. An analysis per organization is reported in the online supplement, showing that a treatment x time effect was found in organization 2 only. Based on the total sample and the model without three-way term, a statistically significant treatment x time interaction was found (*p* = .039). Specifically, the mean outcome difference on intention between treated and control, improved with approximately 0.225 points (*P* = .028; 95 % CI 0.024 – 0.426; *d* = 0.28) at T1 and with approximately 0.039 points (*P* = .720; 95 % CI −0.176 – 0.255; *d* = 0.05) at T2, compared with the baseline difference, see Fig. 5.

Fig. 2, Fig. 3, Fig. 4, Fig. 5 show the time course of the average attitude, social influence, self-efficacy and intention score per treatment condition, at baseline, T1 and T2. The first plot displays the observed outcome (which can be biased due to missingness). The second plot displays the predicted outcome according to the mixed model (which can be biased if the model is incorrect). All plots agree quite well, which provides robustness against bias of either type (missingness, model misspecification).

#### 3.3.2 Qualitative results

##### 3.3.2.1 Changes in attitude

Nurses and trainers reported that most nurses developed a more positive attitude toward activity encouragement behavior through the program. Some nurses noted improved work efficiency, as residents doing more themselves freed up time for other tasks. Families and residents generally responded positively to this approach. However, in some teams caring for residents with a psychogeriatric background, trainers observed lingering doubts among nurses about the suitability of activity encouragement behavior for their target group.

##### 3.3.2.2 Changes in perceived social influence

Most nurses and trainers noted improved collaboration and communication among nurses to enhance resident self-reliance. Teams began setting resident goals, tracking progress, and aligning their approaches. Nurses also held each other accountable when team agreements weren't followed.

*‘’Because of this project, they started the conversation with each other about the resident and why do you do it this way, and how do you do it. How can we ensure that it is all the same so that the resident is challenged by everyone. I thought it was a really positive switch there.’’ [Trainer 2]*

In some teams—particularly the two in organization 3 following trajectory A—motivation was low or team dynamics were poor. Trainers suggested that manager support could have helped, especially in self-managing teams. Trainers and nurses also noted the value of involving other disciplines – which some organizations did – such as an occupational of physiotherapist, who offered fresh perspectives and suggested helpful tools like customized cutlery to support resident self-reliance.

##### 3.3.2.3 Changes in self-efficacy beliefs

Trainers and nurses reported improved nurse self-efficacy in performing activity encouragement behavior due to the program. Initially, some saw only barriers like staffing shortages, time constraints, or perceived mismatches with their target group. Over time, some gained confidence in their own and their team’s ability, though others still hesitated and tended to take over tasks, citing similar challenges as experienced prior to the program. A lack of team motivation or cohesion also made implementation more difficult.

##### 3.3.2.4 Changes in intentions

In general, all trainers and nurses indicated that nurses improved their willingness to enhance their activity encouragement behavior.

*‘‘In all the conversations you noticed that they have been working on it in the meantime and have tried to apply it, from much less automatism and start looking at the resident and how can you stimulate them and things like that more yes, I thought that it certainly had those effects.’’ [Trainer 3]*

As a result, according to the trainers and nurses, the program led to various changes in practice, such as residents preparing their own sandwiches, washing and dressing (partly) independently, combing their hair and shaving, going for a walk and getting the newspaper themselves.

### 3.4 Contextual factors

#### 3.4.1 Facilitators

Trainers did not log facilitating factors for program execution, but in interviews, trainers and nurses identified contextual factors that eased program implementation and providing activity encouragement behavior. Nurses reported that in some organizations (apart from organization 3), a policy regarding activity encouragement behavior was already present. Because of this, the topic was already frequently discussed in staff meetings and the program was viewed as an extra boost to support the policy. Many nurses found family member support helpful in promoting activity encouragement behavior. Trainers and nurses highlighted a motivated, well-functioning team as key to program delivery and providing activity encouragement behavior. Trainers also noted that the program’s clear structure, instruction manual, and interactive nature made implementation easier and more valuable.

*‘’Every training was built up a bit the same, starting with introduction, purpose of the session, that was very nice, it also continues on and reflects back to the previous session and the practical assignments. I think in terms of structure and content, the assignments were very good I think, very interactive.’’ [Trainer 5]*

#### 3.4.2 Barriers

In contrast to the trainers not reporting facilitating factors in the logbooks, they did describe a few barriers. In some cases, the trainers indicated low attendance and time constraints as barriers to optimally provide the program in practice. In addition, in the interviews, nurses and trainers also mentioned some contextual barriers for adequately providing activity encouragement behavior. Staffing shortages and a lack of time were put forward most frequently which led to taking over activities from the residents.

*‘’We know that we have to let people move but you also know, when there is stress and when there is time pressure then you just let that go, I just think that's the pity of everything and that will always remain in healthcare because you are always struggling with staff shortages, at least in the 15 years I've been in healthcare.’’ [Nurse 2]*

One nurse indicated that some family members of their residents were not supportive of the program, because they were afraid the residents’ safety would be at risk (e.g. increased fall risk).

As the program was provided during the COVID-19 pandemic, this caused some issues and resistance in some teams according to the trainers and nurses. For example, several sessions could not be provided because of the national measures, and some nurses did not find the timing of delivery appropriate. One nurse indicated that if the program had been provided after COVID-19, the team would have been more positive about the program. Additionally, not having manager support was viewed as a barrier, especially in the two self-managing teams.

## 4 Discussion

The aim of this process-evaluation study was to evaluate the implementation, mechanisms of impact and contextual factors influencing the implementation and outcomes of the SELF-program.

Nurses and trainers were highly satisfied with the program, particularly valuing its interactive content. The program was implemented with high fidelity, reflected by 90 % of all sessions completed. On average, 60 % of all potential participants attended the sessions, however, attendance rates varied between organizations and declined over time. The program resulted in improvements in nurses’ attitudes, perceived social influence, self-efficacy beliefs and intentions towards performing activity encouragement behavior, of which most were statistically significant, corresponding with small to medium effect sizes. These results were endorsed by data derived from focus-group interviews with trainers and nurses. Time constraints, staffing shortages and lack of motivation were put forwards as factors leading some nurses to still take over tasks. The presence of policy, family member support, motivation of nurses and the interactive approach facilitated program implementation and outcomes. Barriers to program implementation and outcomes included staffing shortages, low program attendance, time constraints, lack of manager support, and delivery of the program during the COVID-19 pandemic.

Despite in- and external barriers such as staffing shortages and COVID-19, the program was largely carried out as planned with no major deviations from protocol, with attendance rates averaging 60 %. Studies addressing the implementation and reach of similar programs are scarce. A recent process-evaluation of a Dutch reablement training program for nurses, demonstrated comparable results regarding program implementation and satisfaction and attendance rates (Rooijackers et al., 2021). Still, in our study 40 % of the potential audience did not attend the training sessions. Although given reasons seem plausible, and despite these are comparable to those reported in previous research, it is evident in future programs to increase attendance. Potentially also the COVID-19 and related local measures, additional illnesses and staffing shortages may have caused the attendance to be lower compared to conditions without COVID-19 (Maltezou et al., 2023). The decline in attendance over time is another concern, however, as addressed by trainers and nurses, the sessions sometimes were repetitive, and the number of sessions could potentially be reduced. In addition, the SELF-program was tailor-made by offering trajectory A or B following the kick-off session. Although this is an element highly recommended by literature as tailored programs and materials are more effective and relevant to their intended audience (Kreuter and Wray, 2003), some differences in program appreciation and attendance were observed. The average attendance was approximately 10 % lower in those teams following trajectory A. Moreover, session 1, which only took place in this trajectory, was rated with the lowest satisfactory score and found to be too repetitive. As motivation to enact activity encouragement behavior was already considered lower in those teams appointed to trajectory A compared to trajectory B, this repetition early in the trajectory may have caused additional lack of motivation to prompt a behavior change and attend subsequent sessions. Despite the SELF-program being developed as an interactive training course and designed to match the needs of teams in various motivational phases, the supposed repetition in session 1 could perhaps be replaced. For example, by incorporating an additional practice-oriented actor session, which was highly valued by the nurses and has shown to stimulate self-refection; an important change mechanism early in the behavior change process (Vluggen et al., 2021; Smeets et al., 2020). This may not only prompt ownership in their own change process but also enhance their motivation and willingness to enact activity encouragement behavior. Last, the combination of being appointed to trajectory A, the absence of manager support and policy regarding activity encouragement behavior, as well as the program not being mandatory, may have added up to the decision of two teams in this organization to discontinue the training. Yet, two other teams within this organization appointed to trajectory B completed the training, potentially demonstrating that they indeed showed sufficient motivation initially (Stausholm et al., 2021). It seems that the participants following the two trajectories were quite equally satisfied with the program. A potential explanation may be that the nursing teams indeed were appointed to the right trajectory – in which they had a key role decisional role – making them satisfied with what they received (Graham-Dickerson et al., 2013). Still, as especially manager support and policy are considered key prerequisites, it seems evident for an organization to arrange these conditions before the implementation of a training program, especially for those teams lacking initial motivation to change (Vluggen et al., 2021).

Despite similar results on implementation parameters, the program studied by Rooijackers and colleagues and other similar programs demonstrated no effectiveness, while the SELF-program was effective in improving nurses’ activity encouragement behavior as well as mechanisms believed to mediate this behavior (Lee et al., 2019; Rooijackers et al., 2022; Buma et al., 2022). However, it should be noted that studies examining the effectiveness of Function Focused Care or Reablement programs often use distinct outcome measures, implying comparisons are difficult and should be made with caution (Buma et al., 2022). The SELF-program was based on several lessons learned and implications from previous programs with similar aims, potentially explaining its effectiveness (Vluggen et al., 2021). For example, a key lesson incorporated in the SELF-program was the application of a sequential dual behavior change process, as opposed to other programs that directly focus on equipping nurses to induce a behavior change among residents. This sequential dual behavior change process primarily aims to increase nurses’ own awareness, motivation and willingness to encourage self-reliance and activity in residents. In doing so, the SELF-program builds on integrative behavior change theory which assumes that a person’s attitude, perceived social influence, self-efficacy beliefs, and intention to perform activity encouragement behavior are the mediating processes that explain behavior change (de Vries, 2017; Resnick et al., 2004). These concepts are at the core of the program, and translated into interactive working methods using effective behavior change techniques (Bartholomew et al., 2016). By targeting these concepts in a sound manner, the program was not only able to improve these mechanisms, but also the primary target behavior of the program, i.e. activity encouragement behavior. Although the effect evaluation revealed a robust change in nurses’ activity encouragement behavior, demonstrating a sustained statistically significant effect over time, the effect of the program on mechanisms of impact showed divergent results. Attitude, intention and social influence statistically significantly improved at T1, of which only the effect of social influence was sustained at T2. Self-efficacy only first statistically significantly improved at T2. It is possible that improvements in nurses’ attitudes, perceived social influences and intention became apparent directly after the program (T1), while building their confidence in performing activity encouragement behavior under certain circumstances took time, experience and vicarious learning (Bandura and Walters, 1977). For example, the team approach applied in the training ensured the whole team worked together in improving their activity encouragement behavior. As nurses started to experiment with activity encouragement behavior in practice and simultaneously saw their colleagues also making this change, this may explain that changes in self-efficacy only became apparent over time (Zamani-Alavijeh et al., 2019). Although self-efficacy improved significantly, still certain key circumstances were prone to low self-efficacy and leading nurses to revert to old patterns, such as time constraints, staffing shortages and lack of (collegial) motivation. These situations are well known and recurrent issues in healthcare nowadays and require specific attention in future versions of the program, for instance by means of clear coping planning strategies (van Sambeek et al., 2023; de Vries, 2017).

Several contextual facilitators and barriers of program implementation and its outcomes were exposed through this study. These facilitators and barriers can roughly be divided into in- and external factors which may also relate to the influenceability of these factors. It has been emphasized earlier that organizational policy and the presence of manager support are key preconditions for the successful implementation of programs and related outcomes (Smidstra et al., 2025; Vluggen et al., 2021). Team managers play a crucial role in translating policy into practice and facilitating behavior change. Not only should they support the transition in terms of being visible, interested and involved, they also could deal with time constraints, e.g. by providing nurses with the required time to undergo a behavior change at first. Last, they have an important role in the sustainability of achieved outcomes. As shown in this study, effects of the program were found on all mechanisms known to impact activity encouragement behavior. However, apart from self-efficacy, the effects became smaller or even statistically insignificant over time. Although the effect-evaluation demonstrated that the effect of the program on activity encouragement behavior was sustained over time, six months after program completion, this effect at T2 was smaller than the effect directly after program completion. It is known in general that program effects decrease over time, and although this was not necessarily the case for the SELF-program, it is evident to keep it in the spotlight, for example by addressing it regularly in team meetings (Rabiee and Glendinning, 2011; Bailey et al., 2020). Beyond management support, this study highlights the importance of a shared ‘Reablement’ vision among all individuals involved in resident care. Nurses and trainers emphasized the added value of involving other disciplines, such as occupational and physiotherapists, as a multidisciplinary approach offers diverse perspectives and creates cumulative value (Hjelle et al., 2016; Gustafsson et al., 2019). Future implementations should therefore engage multiple disciplines. In addition to formal caregivers, involving family members is crucial for a successful care transition. While past studies identified family resistance as a barrier (Resnick et al., 2006; Resnick et al., 2008; Rooijackers et al., 2021) nurses in this study more often reported supportive families, which facilitated activity encouragement. For hesitant family members, a tailored approach—similar to that used with nurses—may help foster acceptance and support of activity encouragement behavior.

Finally, several factors appear to explain why multiple process-evaluation parameters were less favorable in organization 3, relative to the other organizations. Overall, program attendance (reach), the number of sessions completed (dose delivered), and the numerical ratings for most individual sessions (dose satisfaction) were lowest in this organization. Attendance was not mandatory, the organization operated through self-managing teams without a team manager, no policy regarding activity-encouragement behavior was in place, and poor team dynamics and low motivation may have further contributed to these outcomes. Literature consistently identifies these factors as key prerequisites for successful implementation, and their collective absence within this organization may explain the less favorable process-evaluation outcomes observed (Vluggen et al., 2021).

### 4.1 Strengths and limitations

This study has several strengths. Using the Medical Research Council framework allowed for a comprehensive understanding of implementation, impact mechanisms, and contextual factors, while promoting consistency in process evaluations, as urged by literature (Liu et al., 2019). The SELF-program was theoretically grounded, supporting analysis of how impact mechanisms evolved over time (Bugge, 2024). Additionally, combining qualitative and quantitative data provided a well-rounded view of the evaluation parameters. However, some limitations should be noted. Focus group participation was voluntary, leading to a small, potentially biased sample of highly motivated individuals. Furthermore, no nurses from one organization participated, limiting insights into why the program was not completed in some of its teams.

## 5 Conclusion

Nurses and trainers were highly satisfied with the program, the program was implemented with high fidelity, and reasonable attendance. Additionally, the program led to improvements in nurses’ attitudes, social influences and self-efficacy and intention to perform activity encouragement behavior. Although program implementation and outcomes were facilitated by various factors, several in- and external barriers hampered implementation and outcomes such as time constraints, staffing shortages, motivation and COVID-19. The program and its implementation could therefore benefit from minor refinements, as well as an improved attendance. Hereafter, considering the results of this thorough process-evaluation as well as the positive results of the program on nurses’ activity encouragement behavior, a widespread implementation is recommended.

## CRediT authorship contribution statement

**Michel Bleijlevens:** Writing – review & editing, Methodology, Investigation, Conceptualization. **Janneke de Man-van Ginkel:** Writing – review & editing, Investigation, Funding acquisition, Conceptualization. **Gerard van Breukelen:** Writing – review & editing, Validation, Methodology, Formal analysis, Conceptualization. **Sandra Zwakhalen:** Writing – review & editing, Supervision, Project administration, Funding acquisition, Conceptualization. **Lotte Hermens:** Writing – original draft, Methodology, Investigation, Formal analysis. **Silke Metzelthin:** Writing – review & editing, Supervision, Funding acquisition, Formal analysis, Conceptualization. **Getty Huisman-de Waal:** Writing – review & editing, Methodology, Funding acquisition, Conceptualization. **Stan Vluggen:** Writing – review & editing, Supervision, Investigation, Conceptualization.

## Declaration of competing interest

The authors declare that they have no known competing financial interests or personal relationships that could have appeared to influence the work reported in this paper.

## Appendix Supplementary materials

Image, application 1
