# Supplementary material for: Process-evaluation alongside a cluster-randomized trial examining the effectiveness of the ‘SELF-program’ on nurses’ activity encouragement behavior
Source: Int J Nurs Stud Adv. 2026 Mar 16;10:100525. doi: 10.1016/j.ijnsa.2026.100525 (PMC13059108; doi:10.1016/j.ijnsa.2026.100525)
Supplement: Supplementary file 1 [file mmc1.docx]

# Online Supplement 1:

***Social influence***

In organization two, the mean outcome difference on the social influence scale 1 = totally disagree to 5 = totally agree) between treatment and control improved with approximately 0.498 points (p < .001) at T1 and with approximately .377 points (p = .003) at T2, compared with the baseline difference. In terms of Cohen’s effect size as defined for mixed-regression, these two effects were, respectively, *d* = .70 at T1 and *d* = .54 at T2.

***Intention***

In organization two, the mean outcome difference on intention (1 = totally disagree to 5 = totally agree) between treatment and control improved with approximately 0.547 points (p < .001) at T1 and with approximately 0.328 points (p = .042) at T2, compared with the baseline difference. In terms of Cohen’s effect size as defined for mixed-regression, these two effects were, respectively, *d* = .70 at T1 and *d* = .38 at T2.
